# Commentary: To Be Oats or Not to Be? An Update on the Ongoing Debate on Oats for Patients With Celiac Disease

**DOI:** 10.3389/fped.2020.573151

**Published:** 2020-11-11

**Authors:** Ronald D. Fritz, Yumin Chen

**Affiliations:** PepsiCo R&D Data Science & Analytics, Barrington, IL, United States

**Keywords:** Celiac disease, gluten-free diet, oat, gluten-free oat, gluten contamination, gluten containing grains

Spector Cohen et al. recently reviewed the appropriateness of oats as part of a gluten-free diet (GFD) for celiac disease (CD) patients (1). They conclude that, “Inclusion of oats in a GFD might be valuable due to their nutritional and health benefits and improvement of food variety” but advise that, “Nevertheless, since the potential for sensitivity/toxicity exists, oats should be added with caution to a GFD.” We agree that oats can provide nutritional and quality-of-life benefits to CD patients but believe that the reason for caution in their GFD inclusion should be viewed from a different perspective than presented. That perspective is based on our research regarding gluten contamination in oats, its removal, and GF compliance testing to define oats as pure, which potentially explains inconsistent results in CD patient oat feeding studies. We believe this differing perspective can positively influence decision making on whether and how to include oats in a CD patient's diet.

The authors suggest the risk of oat inclusion in CD patient's diets is 2-fold, one relating to a commonly held view that some CD patients are sensitive to pure oats. As the authors cite, clinical results have been inconsistent in this regard, but nevertheless, a collection of important feeding studies have obtained results suggesting this may be the case. A second risk relates to cross contamination potential. The authors infer that this is primarily due to shared production lines with wheat products.

A collection of recent research, however, sheds new light on these risks:

1. **Whole-grain oats are prone to a unique contamination mode** (2). This is caused by gluten-containing grains (GCGs) of wheat, barley, and rye found to regularly contaminate oats (3–6). These GCGs remain intact to the spoon in whole grain products like oatmeal and, thereby, present a concentrated pill-like gluten dosing sufficient to illicit adverse clinical responses in a sizable portion of the CD population (7–9).
2. **Industry has overlooked the subtle, yet profound, implications of this GCG contamination circumstance**, mis-assessing GF oats as pure when they are not, especially on a serving size level. Large “in-market” studies suggest one in every few dozen servings of GF-labeled oatmeal contains a GCG (2, 10). This includes oats produced under a “purity protocol” (i.e., produced under strict farm, transportation, and processing requirements).
3. **To account for the “needle in the haystack” circumstance, which GCG contamination presents, special sampling and analytical assay approaches are necessary** to attain “serving level” GF compliance (i.e., where rate of servings ≥ 20 ppm gluten is very rare). Highly capable approaches have now been developed and published (11, 12).
4. **Some industries have applied these new sampling and analytical methods to their GF oat processes**, attaining an average outgoing quality limit = 1 in 20,222 (i.e., maximum potential outgoing serving rate containing a GCG). This compares to one in every few dozens containing a GCG when employing legacy sampling and testing approaches (13).

These research findings highlight the extent to which oats suffer from GCG contamination and the resulting difficulty this unique type of contamination creates in assessing oat servings as GF. It shows the consequences of overlooking this as well, which is believed universal till recently. These discoveries suggest pure oats should be defined by how GCG-based gluten contamination is removed and then assessed in compliance to GF regulation at the serving size level. This notion deserves consideration when weighing the risks the authors cite.

For the risk related to some CD patients potentially possessing a sensitivity to oats, the notion of how “pure oats” are practically defined prompted us to explore whether oats used in studies prior to these findings were truly pure. Is it possible that feeding studies inadvertently provided oat servings to study subjects, assuming them pure, but they were intermittently contaminated with GCGs? Could this explain inconsistent clinical outcomes to date as well?

A recent theoretical analysis was performed following this premise (14), where out of 433 oat feeding studies considered, 12 were found suitable for meta-analysis and statistical comparison of suspected oat purity vs. subjects encountering adverse reactions. These 12 studies were mentioned by Spector Cohen et al. since they are key contributors to the oat safety for CD patient debate. For these 12 studies, it was found that three used straight commercial oats known to be highly GCG contaminated (3–6), eight used GF oats under a 200-ppm gluten maximum (the standard of the time) and one used oats under today's 20-ppm maximum (but prior to recent GCG revelations). Using cited study dosages and published contamination rates for these three oat “purity classes,” adverse clinical and morphological reaction rates were regressed against theoretical gluten doses (due to presumed GCG contamination). A statistically significant positive trend was uncovered with higher adverse clinical reaction rates coming from studies with presumed less pure oats used; *P* = 0.0006. Adding one more supporting datum to this trend, a recent double-blind, randomized, placebo-controlled trial shows that feeding well-controlled pure nonreactive (i.e., gluten-free) oats are safe to children with celiac disease (15).

So, since conflicting clinical feeding study outcomes exist, and adverse study outcomes correlate strongly with likely oat purity, we believe the jury is still out regarding whether some CD patients possess a sensitivity to “pure” oats. It is possible that the inconsistent study outcomes are explained by the differences in study oats rather than the differences in study subjects. This would not be settled until clinical trials using today's high purity oats are conducted, a prudent next step. In the meantime, we believe that the risk to CD patients reacting negatively to pure oats should be viewed in the context of these findings.

Regarding the risk which CD patients face due to oat contamination, this is clearly real and believed understated by the authors, but great strides have been made and statistically verified that “serving level compliance” GF oats are now available.

In summary, we believe the caution the authors appropriately recommend for inclusion of oats in CD patient's GFD should be exercised from a perspective of oat purity, both from a contamination risk standpoint as well as a potential oat sensitivity one. By doing so, clinicians and CD patients can more appropriately weigh oat's benefits against probable risks, and investigators can pursue an unambiguous conclusion to the “CD patient pure oat sensitivity” debate.

## Author Contributions

Both authors worked on this response and agreed on the final version of this manuscript.

## Conflict of Interest

RF and YC are salaried employees of PepsiCo Inc. or Quaker Foods and Snacks (QFS), a subsidiary of PepsiCo Inc., which funded this research. QFS has a commercial interest in gluten-free foods. The funder was not involved in study design, collection, analysis, interpretation of data, or the writing of this article but did review the article approving it for publication. The views expressed in this manuscript however are those of the authors and do not necessarily reflect the position or policy of PepsiCo Inc.
